# Crack-induced Ag nanowire networks for transparent, stretchable, and highly sensitive strain sensors

**DOI:** 10.1038/s41598-017-08484-y

**Published:** 2017-08-11

**Authors:** Chan-Jae Lee, Keum Hwan Park, Chul Jong Han, Min Suk Oh, Banseok You, Young-Seok Kim, Jong-Woong Kim

**Affiliations:** 1 Display Materials & Components Research Centre, Korea Electronics Technology Institute, Seongnam, 463-816 Korea; 2 School of Advanced Materials Engineering, Chonbuk National University, Deokjin-Dong 664-14, Jeonju 561-756, Korea

## Abstract

Crack-based strain sensor systems have been known for its high sensitivity, but suffer from the small fracture strain of the thin metal films employed in the sensor which results in its negligible stretchability. Herein, we fabricated a transparent (>90% at 550 nm wavelength), stretchable (up to 100%), and sensitive (gauge factor (GF) of 30 at 100% strain) strain gauge by depositing an encapsulated crack-induced Ag nanowire (AgNW) network on a hydroxylated poly(dimethylsiloxane) (PDMS) film. Stretching the encapsulated AgNWs/PDMS resulted in the formation of a percolation network of nanowire ligaments with abundant percolation paths. The encapsulating polymer was designed to adhere strongly to both the AgNW and PDMS. The improved adhesion ensured the resistance of the crack-induced network of AgNWs varied reversibly, stably, and sensitively when stretched and released, at strains of up to 100%. The developed sensor successfully detected human motions when applied to the skin.

## Introduction

Piezoresistive strain gauges are used to measure the strain experienced by an object under an applied force and are typically fabricated by laminating patterned metal foil or wires on an insulating flexible support^1–4^. These devices are widely employed to measure mechanical deformations in various applications including weighing scales, impact sensors, and medical sensors, because of their simple structure and read-out mechanism. Recent advancements in robotics and biomedical devices have led to the vigorous development of new stretchable strain gauges to attain minimally invasive and implantable devices^5–8^. Stretchable strain gauges can adhere or be embedded in various complex surfaces, such as skin, tissue, or organs, without severe wrinkling and buckling. Furthermore, this class of strain gauges can be stretched, compressed, twisted, and deformed into complex, non-planar shapes while maintaining high sensitivity, reliability, and integration^9, 10^.

Various materials have been proposed for use in stretchable sensors and electronic devices, including carbon nanotubes (CNTs)^11, 12^, graphene^13–15^, carbon black^16^, poly(3,4-ethylenedioxythiophene):poly(styrenesulfonate) (PEDOT:PSS)^17^, and metal nanowires^18–20^. Stretchable strain gauges using these materials are typically fabricated from a mixture of the conductive materials and a non-cross-linked liquid polymer, which yields a three-dimensionally networked conductor when the polymer is cured^21^. Stretching the cured composite structure decreases the percolation density, resulting in decreased electrical conductivity. Another method used to produce these devices is to bury the percolated conductive networks at the surface of the cross-linked polymer^22^. Since this approach usually employs metal nanowires or CNTs in very low densities, the fabricated structures are typically transparent; this is an advantage for their use in devices. However, the sensitivity of the strain gauges produced by both these methods is typically lower than those of crack-induced strain gauges.

Ultrasensitive strain gauges based on crack-induced sensor systems have recently been reported to achieve gauge factors (GF) as high as 16,000 at 2% strain, which is significantly higher than those of reported composite structures^23, 24^. In these crack-induced sensors inspired by the geometry of a spider’s slit organ^23^, the metallic nanoscale cracks formed in a polymer layer are disconnected and reconnected with adjacent crack junctions when strain is applied, which enables subtle changes in resistivity to be detected in response to external forces^24^. The electrical resistance of a metal layer with some artificially formed cracks experiences an abrupt drop from a finite value when the edges of the cracks are in contact, to zero when they disconnect. In this case, the high strain sensitivity originates from the rare yet large gap-bridging steps on opposite edges of the artificially formed crack^23^. However, since these sensors have employed the metal thin films such Pt for crack formation, the maximum strain that can be withstood by the surface of these sensors is limited to ~2% by the low fracture strain of the metals.

Herein, we demonstrate that sensors based on crack junctions artificially formed on a percolated AgNW network/poly(dimethylsiloxane) (PDMS) can simultaneously attain high sensitivity and stretchability. We used a stretchable polymer which is compatible to both the AgNWs and PDMS for encapsulating the device in order to improve its reversibility, reproducibility, and durability. The fabricated strain gauge was highly sensitive, highly stretchable and optically transparent, which frees the device from problems of privacy when used to detect human motion. The fabricated strain gauge could successfully detect strains induced by muscle movements on the skin of a proximal interphalangeal joint and a human face.

## Methods

### Materials and Synthesis

Materials and synthesis for polyurethane urea (PUU) were described in our previous studies^25, 26^. A Sylgard 184 elastomer kit was obtained from Dow Corning, USA. Pure PDMS was prepared by mixing the base and curing agent with a weight ratio of 10:1.

### Fabrication of strain gauge

**Figure 1:**
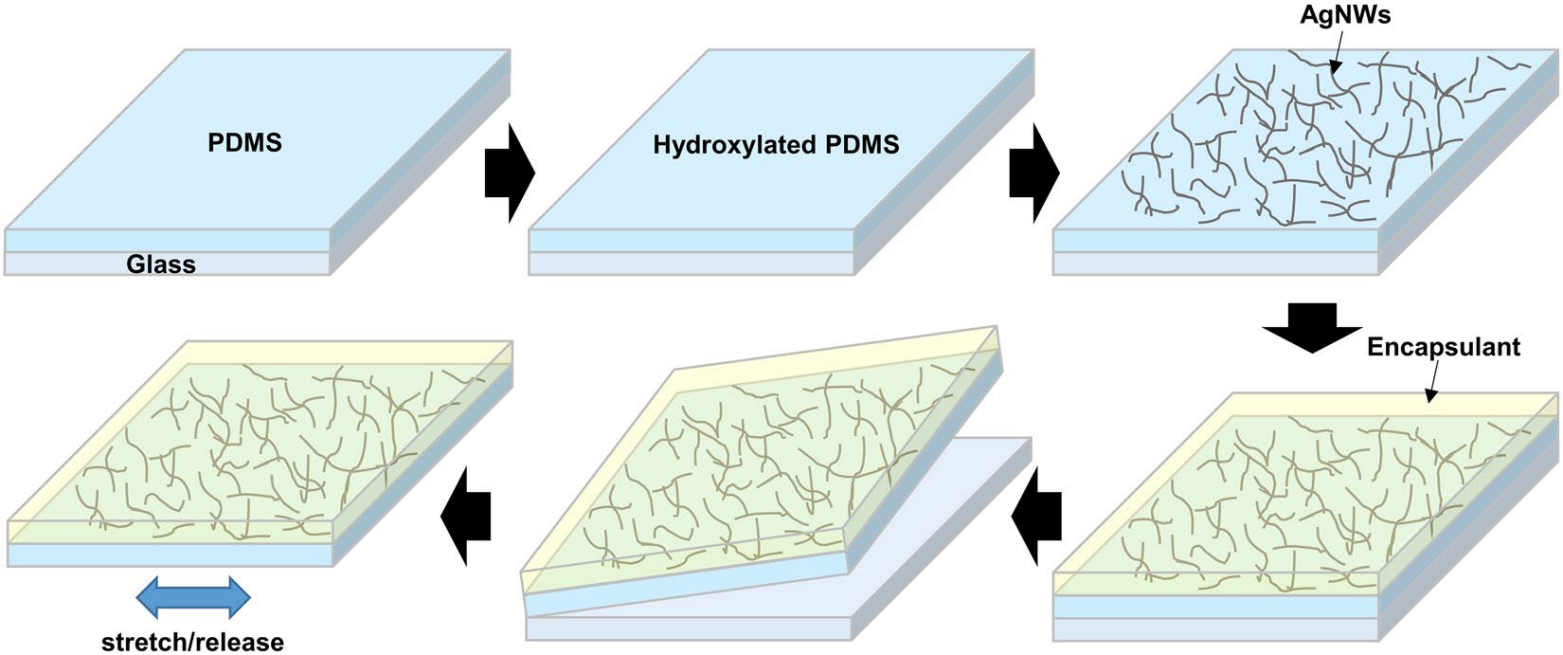
Schematic illustration of the fabrication of crack-induced strain sensor.

The fabrication of the stretchable strain gauge is schematically illustrated in Fig. 1. A glass substrate was first cleaned using detergent, de-ionized water, isopropanol, and acetone. Pure PDMS was spin-coated onto the glass, degassed, and thermally cured at 70 °C for 12 h. The thickness of the PDMS layer after curing was around 150 μm. Oxygen plasma treatment was used to form hydroxyl groups on the PDMS film. The O_2_ gas flow rate, gas pressure, power, and treating time were controlled to be 35 mL/min, 20 Pa, 50 W, and 120 s, respectively. A solution of AgNWs dispersed in isopropanol (0.7 wt.%; Nanopyxis Ltd., Korea) was spin-coated onto the PDMS film and heated on a hot plate at 60 °C for 10 min to remove any remaining solvent from the coated layer. The synthesised encapsulant was spin-coated (spin velocity: 800 rpm, duration time: 60 s, temperature: 25 °C) onto the AgNWs/PDMS film, followed by drying at 25 °C for 30 min to achieve a coating with a thickness of 20 μm. The encapsulant/AgNWs/PDMS sensor was then peeled from the glass and stretched and released at a specific strain to form cracks in the sensor.

### Evaluation of sensor

Field-emission scanning electron microscopy (FESEM; JSM6700F, JEOL Ltd., Japan) was used to investigate the microstructure of the AgNW networks. The optical transmission was also measured using a UV–visible spectrophotometer (V-560, Jasco, Japan), while the sheet resistance (R_s_) was measured using a non-contact measurement system (EC-80P, Napson Corporation, Japan). An automatic stretch-testing machine (Stretching Tester, Jaeil Optical System, Korea) was used to measure the long-term reliability under repeated cycles of stretching. The sensors were stretched and released at a rate of 0.5 mm/s under varying strains to measure the stress-strain behaviour, and the electrical resistance was measured during testing. This test used uniaxial tensile strains of 25, 50, 75, and 100%, and stretched the samples at a cycle rate of 1.2 cycles/min. We also conducted a long-term cyclic test of up to 2500 cycles at 100% strain. The sensor was worn by an adult human to assess the wearability as well as the performance of the sensor under stretching condition at different body locations. Informed consent was obtained from the human subject prior to the experiments. We confirm that the Ethical Committee of the Korea Electronics Technology Institute (KETI) approved all the experiments described in this paper. All methods in this study were performed in accordance with the relevant guidelines and regulations in KETI. More than 20 samples were fabricated, and their sensing performance was evaluated.

## Results and Discussion

**Figure 2:**
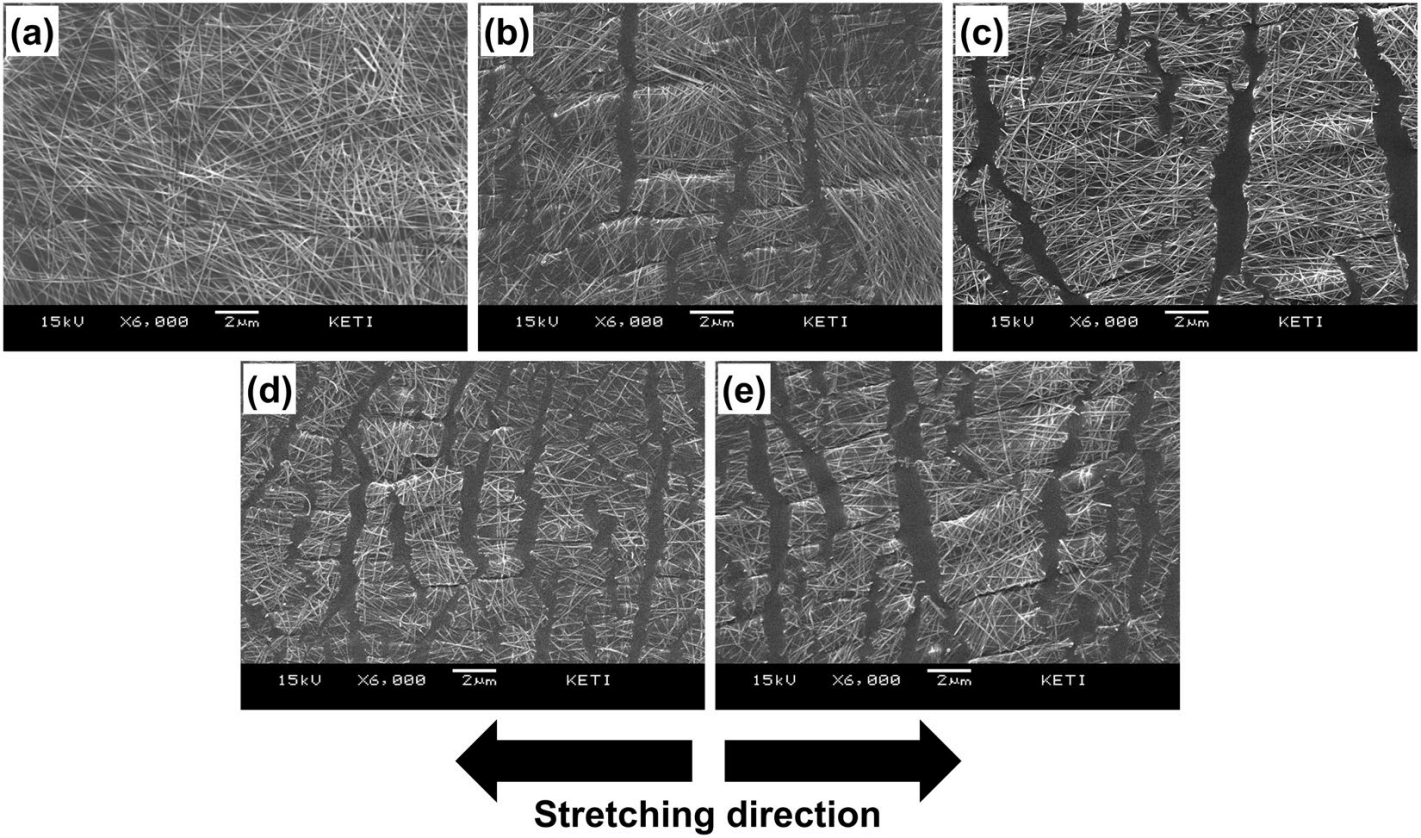
SEM micrographs of AgNWs deposited on hydroxylated PDMS: (**a**) the pristine state, (**b**) the 25% strain stretched state, (**c**) the 50% strain stretched state, (**d**) the 75% strain stretched state, and (**e**) shows an image during stretching at 100% strain.

Since no thorough studies on the mechanism of fracture under large strains of AgNWs deposited on a stretchable substrate have been reported, we investigated the type of failures that might occur in the nanowire network by applying a large strain to AgNWs-based elastomeric electrodes. We first prepared a stretchable transparent conductor by depositing AgNWs on a hydroxylated PDMS sheet using the method illustrated in Fig. 1. We investigated the surfaces of the AgNW electrodes under varying levels of strain using an FESEM, and the results are summarised in Fig. 2. As shown in Fig. 2a, the nanowires were deposited on the PDMS surface, resulting in a porous networked structure. No disconnections of the nanowires, severe aggregation or cracking of the underlying polymer were observed in the FESEM analysis. We note that the electrode was much rougher (root-mean-square roughness (R_RMS_) of around 36 nm) than the embedded structures^27^, implying that some parts of the nanowire networks did not make a stable contact with the functionalized surface of PDMS. Some microcracks were formed in the AgNW forest when we applied a uniaxial strain of 25% to the AgNWs/PDMS, generating an ordered network of nanowire ligaments divided with cracks aligned perpendicular to the stretching direction.

We employed AgNWs to achieve a stretchable strain gauge because the network structure of AgNWs is known to be appropriate for fabrication of flexible and stretchable electrodes based on two key properties: i) the intrinsic durability of individual AgNWs, and ii) the networked structure of AgNW/polymer materials. Greer and Nix explained the first property using the dislocation starvation effect, in which dislocation in the nanoscale crystals can travel only very limited distances before annihilating at free surfaces. Thus, the overall dislocation multiplication rate can be significantly lowered^28^. Gliding dislocations leave the crystal more rapidly than they multiply, thereby decreasing the overall dislocation density. This decrease leads to a dislocation-starved state requiring very high stresses to nucleate new dislocations^28^. The second property arises from a geometrical feature of the AgNWs/polymer electrode, in that much of the stress is relieved by the softer polymer regions when a large strain is induced in a low-density AgNW network. Further, individual nanowires can slide during the stretching and release of the AgNW/polymer composite, providing excellent resistance to stretching fatigue^29^.

While these advantageous properties suggest that a stretchability of up to 100% might be reasonably expected for AgNW-based elastomeric electrodes, the fracture strain range of AgNWs has not been reported to exceed 4%^30^ despite the dislocation starvation effects. Our tests showed that the application of a 5% uniaxial strain to the fabricated AgNWs-based elastomeric electrodes induced separation of the ligament structure and formation of vertically aligned microcracks. These cracks may have originated from the parts of the nanowire network that were not in contact with the functionalized PDMS surface. Figure 2 shows that the width and density of the microcracks increased with an increase in the applied strain, forming either large cracks or a fine ligament network of AgNWs at higher strain levels. The PDMS substrate elongated in a specific direction, forming stable contacts with AgNWs during stretching to a specific level of strain. However, higher strain levels are believed to detach some parts of the nanowire network from the underlying PDMS, causing the crack-like structures in the stretched state.

**Figure 3:**
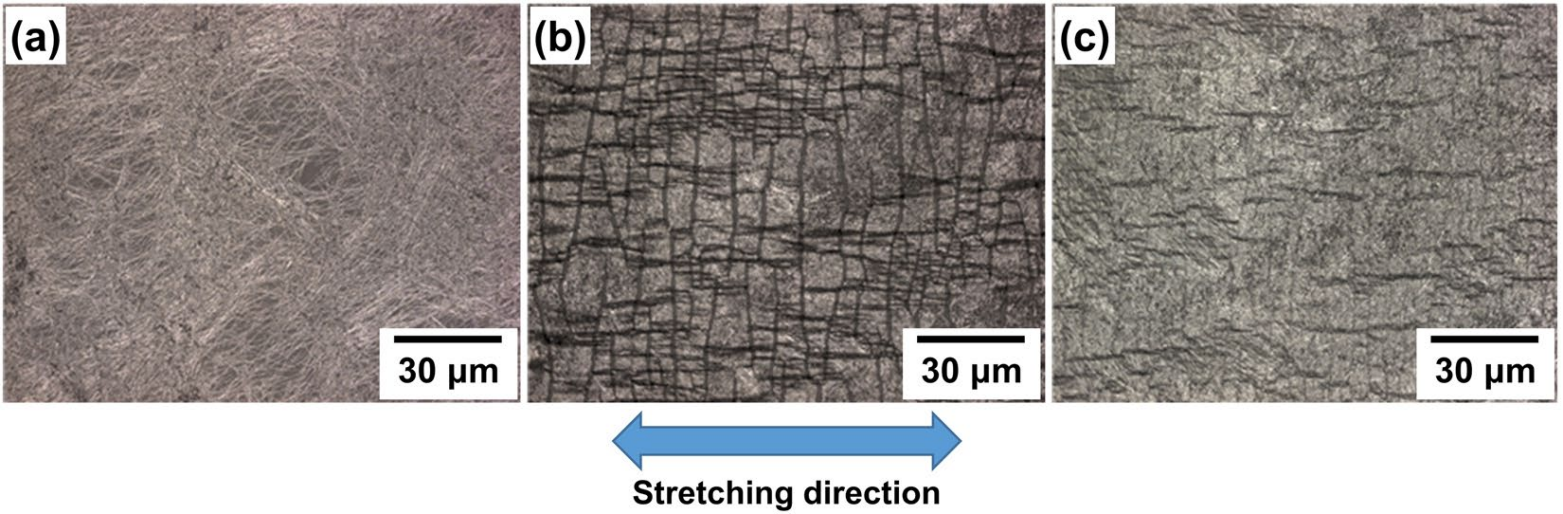
AgNWs deposited on a hydroxylated PDMS film, (**a**) the pristine state, (**b**) under stretching with 100% strain and (**c**) after release.

**Figure 4:**
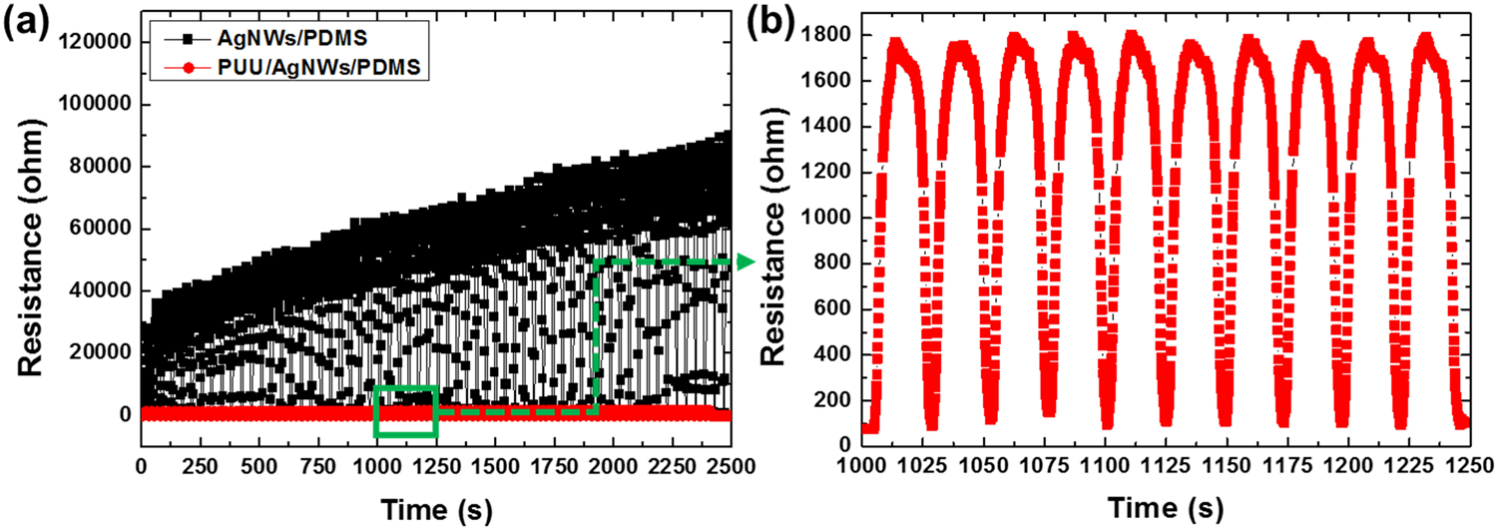
(**a**) Resistance variation of the AgNWs/PDMS and PUU/AgNWs/PDMS sensors measured during stretching and release (strain: 100%) cycles for up to 2500 cycles. (**b**) Show that of the PUU/AgNWs/PDMS in the time range of 1000–1250 s.

The microstructural images shown in Fig. 3 show a cracked nanowire electrode after being stretched and released. The microcracks induced by stretching formed a uniformly shaped pattern spontaneously to release the large induced strains. The separation of the ligaments should increase the electrode’s resistance, but also allows room for it to recover its original shape and resistance, as shown in Fig. 3. The prominent cracks closed after release, implying that the separated ligaments were re-joined. Since the increase and decrease in resistance on stretching and releasing the electrode were expected to be reversible, we induced 100% strain on the fabricated electrode and released. The resistance significantly increased to beyond 100 kΩ after the first stretch, and the resistance after release the strain was significantly higher than the original value; this irreversibility was attributed to the mechanical instability of the electrode (Figure S2). Inverted-layer processing was firstly employed to enhance the stability of the electrode^27^: AgNWs were embedded in the PDMS surface using a previously reported method^31^. The inverted-layer processing has been used to achieve a robust AgNW-based flexible/stretchable electrode by an embedment of nanowires into the surface of a polymer. For this, the nanowire dispersion is firstly deposited onto a preliminary supporting substrate, such as glass, Si wafer or polymer film, followed by overcoating of liquid polymer such as polyimide varnish or PDMS solution, curing and peeling-off from the preliminary substrate. This simple procedure made the nanowires fully buried beneath the surface of the polymer, so that can exhibit an enhanced mechanical stability and surface smoothness. The resistance of the embedded structure was measured during a stretch-release test employing a maximum strain of 100%, as shown in Fig. 4a (plotted with solid black squares). The mechanical stability and reversibility were significantly enhanced, but the maximum resistance continuously increased throughout the test. We investigated the microstructure of the embedded electrodes during the stretching process. The formation of the ligament network was not observed; instead, the nanowires behaved individually, rather than as a body. This behaviour occurred because the individual nanowires all contacted the polymer support. We suggest that individual nanowires were damaged beneath the surface of the PDMS during stretching, leading to the deterioration in the conductivity of the electrodes.

Two important requirements must be satisfied for the application of an electrical conductor in a stretchable sensory system: its conductivity must be reversible, and there must be a large variation in resistance with applied strains. The two systems discussed above fulfilled the second requirement but not the first, because of their insufficient mechanical stability. Therefore, we employed a transparent and stretchable polymer (PUU) to encapsulate the AgNWs/PDMS composite electrode. This polymer was originally designed to enhance the adhesion between AgNWs and PDMS and thereby facilitate the production of a stable and stretchable AgNW/PDMS-based electrode based on them^25, 26^. The presence of carboxylic acid groups in the PUU polymer chain ensured its stable adhesion to AgNWs, and the favourable interactions between AgNWs and these hydrogen-bond donating carboxylic acid groups increased the affinity of the polymer matrix for the AgNWs^25^. Furthermore, the encapsulant could adhere to both the AgNWs and PDMS via hydrogen bonding between the hydroxyl groups of PDMS and the urea (or urethane) of PUU; this adhesion was expected to enhance the interlayer interactions^25^. The high clarity and transparency of the polymer resulted in the encapsulated sensor having a transmittance of ~90.6% (at 550 nm) and R_s_ of 11.01 Ω/□ as shown in Figure S1. The resistance of the electrode (width: 5 mm and length: 10 mm) was measured during stretch/release employing a maximum strain of 100%, as shown in Fig. 4a (solid red circles) and 4b. The resistance increased from 79 Ω and decreased with each stretch and release, respectively, with a negligible change in the maximum resistance over 2500 cycles. We assumed that the ligaments in the AgNWs film formed a percolating network, ensuring electrical continuity. When elastomeric substrates that lacked an encapsulant were stretched, uniform-shape ligaments were formed, as previously discussed, that resulted in a large increase in the resistance during stretching and enhanced the strain sensitivity. However, while the variation of the electrode with stretch/releasing was decreased in the encapsulated sensor, the mechanical stability was greatly enhanced.

**Figure 5:**
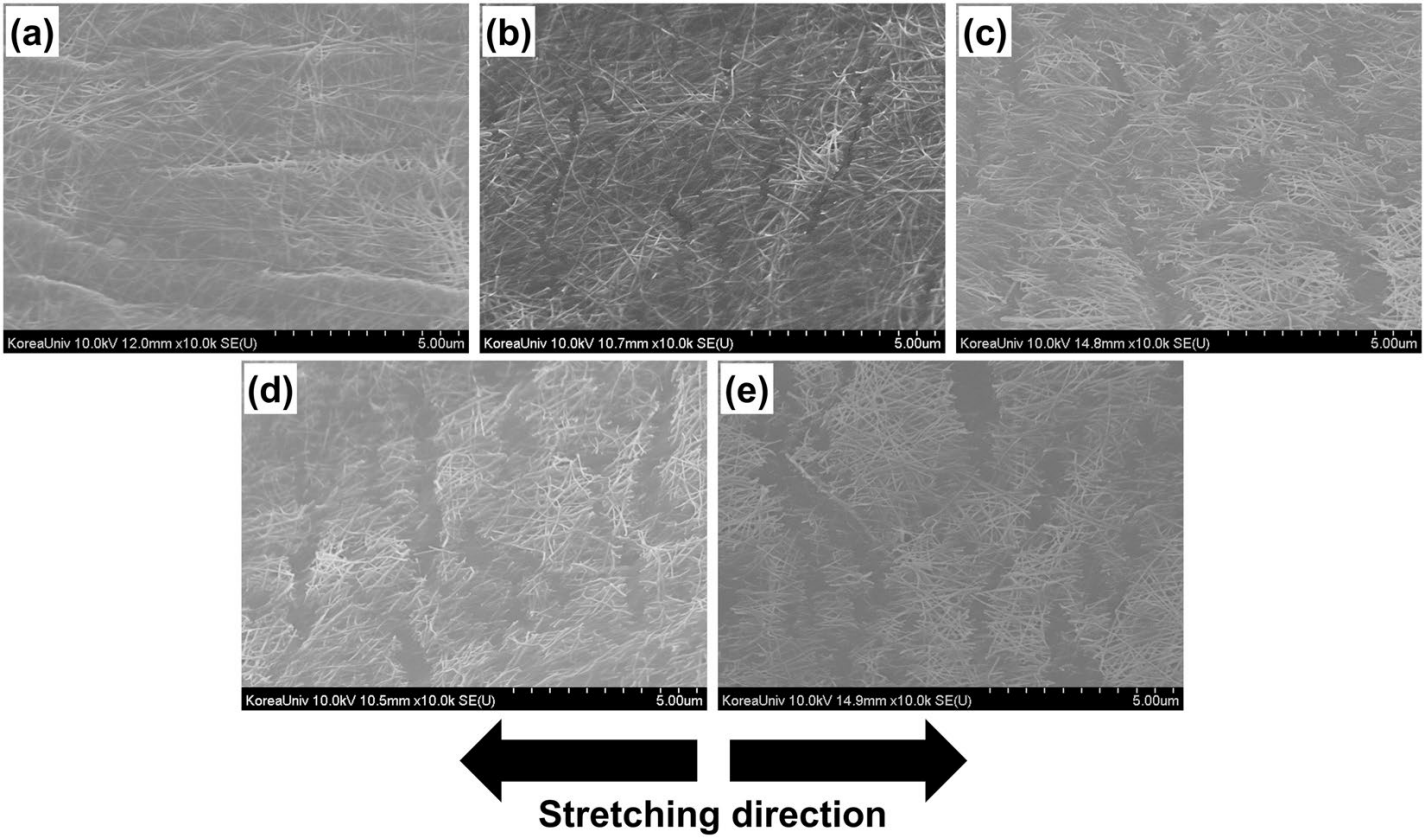
SEM micrographs for encapsulated AgNWs deposited on hydroxylated PDMS: (**a**) the pristine state, (**b**) the 25% strain stretched state, (**c**) the 50% strain stretched state, (**d**) the 75% strain stretched state, and (**e**) the 100% strain stretched state.

**Figure 6:**
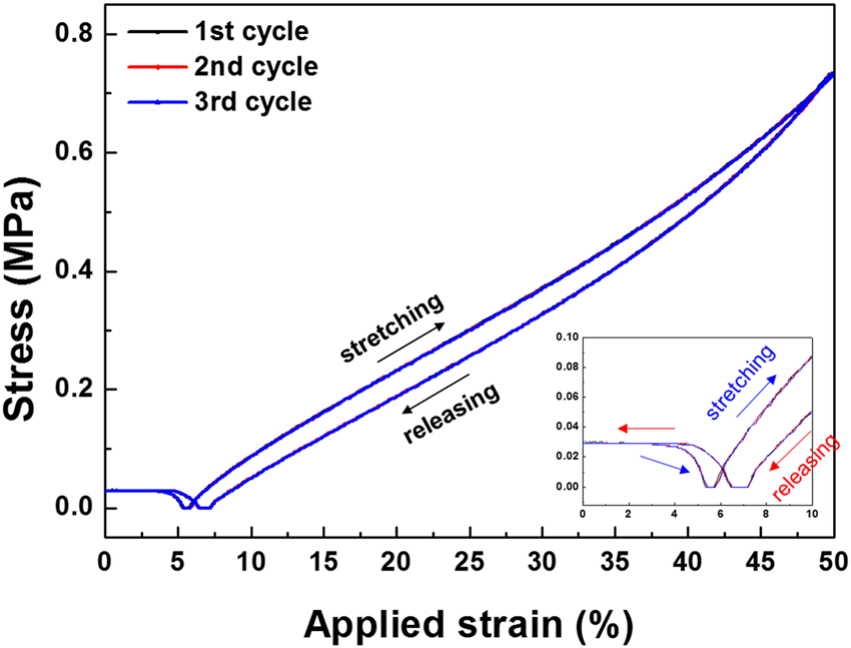
Stress-strain behaviour of the fabricated crack-induced strain sensor.

We investigated the electrodes after stretching with varying strain loads, as shown in Fig. 5, to analyse their strain sensitivity. The formation of a thin polymeric layer on the AgNWs reduced the contrast of the SEM images of the nanowires. As for the electrodes without PUU, the encapsulated nanowires formed a percolated ligament network with microcracks aligned perpendicular to the stretching direction. However, the microcracks were less ordered in the encapsulated electrode, so the percolating paths were more abundant even for the highest applied strain values. The SEM images indicate that the ligaments remained in contact with each other and that the gaps between AgNWs were less prominent. The microstructure was scarcely affected by repeated stretch–release tests, which explains why the dynamic resistance range was smaller, and the encapsulated electrodes were more stable than the bare AgNWs/PDMS electrodes. As the stretching increased, the protected ligament network twisted and was deflected out of the plane of the sensor^32^. Thus, a large elongation of the elastomeric substrate induces only weak elastic strains in the AgNW network. We also investigated the stress–strain behaviour by employing continuous stretch-and-release testing without an interval between cycles. Figure 6 shows the hysteresis curve for the encapsulated electrode, indicating that the hysteresis curves completely converged and that reversibility was achieved. However, a demerit of the sensor was also found in the hysteresis curve: the sensor was not properly stressed in the low strain range (0 to 7%), possibly originated from the residual stresses generated during the crack-making pre-stretching process. This tendency was also found in the graph for the resistance of the sensor measured with the applied strain (Figure S3), in that the sensitivity of the sensor was rather low in the low strain region.

Considering the high sensitivity in high strain range (30 GF at 100% strain) with the low performance in low strain region, our sensor is suitable for detecting large motions of stretchable objects such as movement of human body joints or change in the facial expressions, while may not be acceptable for detecting subtle motions or movements such as heartbeats or dilatation of the pupil. The electrode’s transparency and stretchability ensure that the performance of the sensor is competitive with previously reported electrodes^33–35^. Amjadi *et al*. fabricated a stretchable strain sensor based on an AgNW and PDMS elastomer nanocomposite in a sandwich structure^33^. The sensor was stretchable by up to 70% and suitable for application in motion detection. However, the highly dense nanowire networks rendered the electrode opaque, and it was relatively insensitive (14 GF) compared to our electrode. Yao and Zhu fabricated a piezocapacitive strain gauge using AgNWs as electrodes (conductors) and Ecoflex as a dielectric^34^. Their sensor showed a linear response to tensile strains of up to 50%, and a bilinear response to pressure with a low GF of ~0.7. Further, their device was also not optically transparent because of the use of a highly dense AgNW network. Lee *et al*. reported a sensor that used the change in the electrical resistance of percolated Ag nanoparticles on a PDMS film caused by the opening/closure of micro-cracks under mechanical deformation^35^. Although they employed the crack-induced approach, their sensor could be stretched by up to 20%. However, the GF of their sensor was low (2.05 GF at 20% strain), and it was also opaque. To the best of our knowledge, our electrode has a higher sensitivity at high strain range (30 GH at 100% strain) than any reported transparent (higher than 90% at 550 nm wavelength) and stretchable (up to 100% strain) strain gauges. We here demonstrate its functionality as a motion sensor by investigating its ability to detect both the motion of the proximal interphalangeal joints and changes in facial expression.

**Figure 7:**
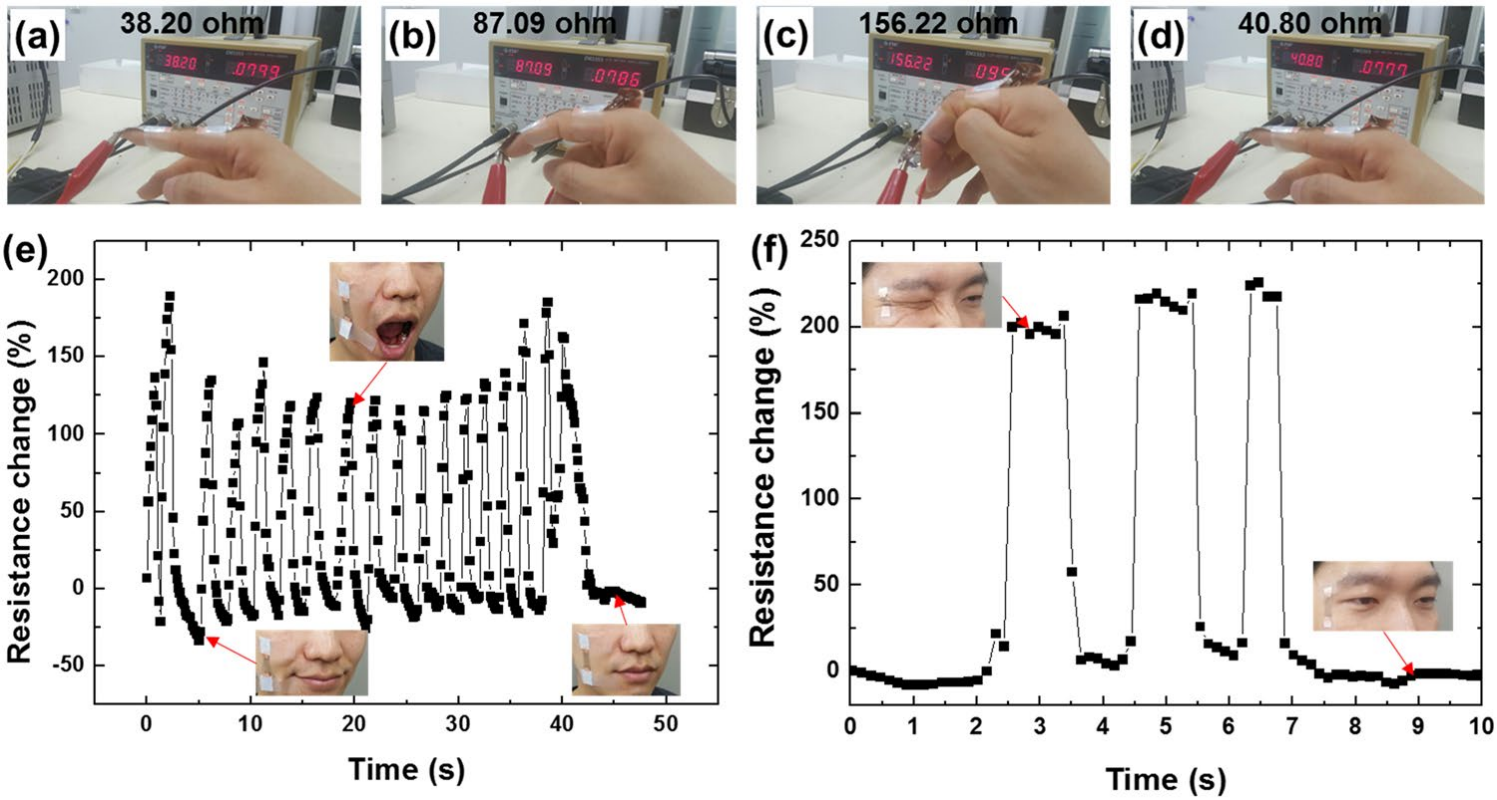
Resistance of the crack-induced sensor attached to the skin (**a**–**d**) around a proximal interphalangeal joint, (**e**) around a human cheek, and (**f**) a lateral area of a human eye.

Figure 7a–d show the sensor being used to detect the motion of a finger joint. The sensor was affixed to the joint when the finger was fully bent, so that good adhesion could be attained without forming any pores or discordant wrinkles under the stretched state. The resistance across the elastomer electrode was measured as the finger moved. We found that the resistance varied very sensitively with the motion of the finger. The sensor could detect the finger joint bending forward and backwards, exhibiting clearly distinguishable changes in resistance. We also affixed the sensor to a human cheek and monitored in real time the resistance change induced in the sensor by the mechanical motion of the skin as the subject opened and closed his mouth several times (Fig. 7e). The resistance varied sensitively with the motion of the facial muscles, and the sensor could even detect subtle differences in expression near the mouth (see the resistance and expression at 5 s and 45 s). We also measured the movement of the lateral skin of a human eye induced by winking (Fig. 7f). The resistance of the sensor sensitively and reproducibly varied with the winking motions, implying that the fabricated sensor system can be useful for motion detection in diverse parts of the human body.

## Conclusion

We successfully fabricated a highly stretchable and transparent strain sensor based on crack-induced AgNW networks that were encapsulated by a stretchable and transparent polymer. The encapsulating polymer was selected for its compatibility with AgNWs, and its ability to form hydrogen bonds between the carbonyl groups of the poly(vinylpyrrolidone) layer at the surface of AgNWs and the carboxylic acid groups of the polymer. Furthermore, hydrogen bonding between the hydroxyl groups of the plasma-treated PDMS and urea (or urethane) ensures that the encapsulant can simultaneously adhere to both the AgNWs and PDMS, which is expected to enhance the interlayer interactions. The encapsulating polymer was deposited on an AgNWs/PDMS electrode to enhance the mechanical stability of the electrode. The nanowires formed a percolated network of nanowire ligaments with abundant percolating paths in response to the stretching and release of the encapsulated AgNWs/PDMS electrodes. The resistance of the electrodes sensitively increased and decreased upon stretching and release, respectively, with negligible hysteresis and high GF (30 GF at 100% strain). To the best of our knowledge, this is the first report of a transparent strain sensor demonstrating both high sensitivity and large stretchability up to 100% strain. The excellent sensing performance of the fabricated transparent sensor was demonstrated by the detection of muscle-induced strains on the skin of a proximal interphalangeal joint and a human face.

## Electronic supplementary material

Electronic Supplementary Information
