# Supplementary material for: Crack-induced Ag nanowire networks for transparent, stretchable, and highly sensitive strain sensors
Source: Sci Rep. 2017 Aug 11;7:7959. doi: 10.1038/s41598-017-08484-y (PMC5554203; doi:10.1038/s41598-017-08484-y)
Supplement: Supplementary file 1 — Electronic Supplementary Information [file 41598_2017_8484_MOESM1_ESM.pdf]

Supplementary Information

Crack-induced Ag nanowire networks for transparent, stretchable, and highly sensitive strain sensors

Chan-Jae Lee, Keum Hwan Park, Chul Jong Han, Min Suk Oh, Banseok You, Young-Seok Kim, and Jong-Woong Kim

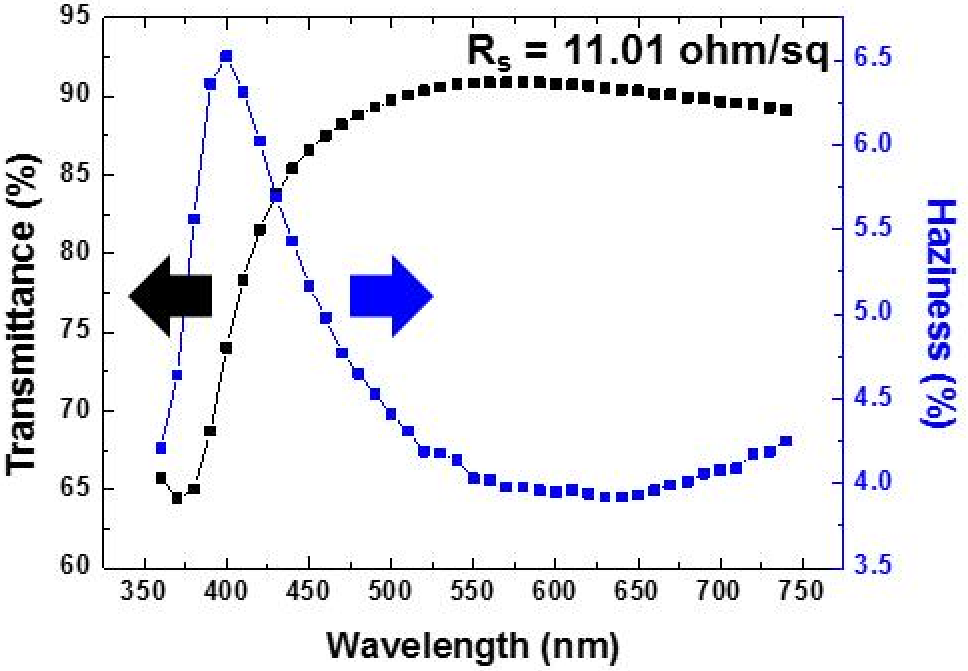

Figure S1. Optical transmission and haziness spectrum of the fabricated sensor (R_S: 11.01 ohm/sq).

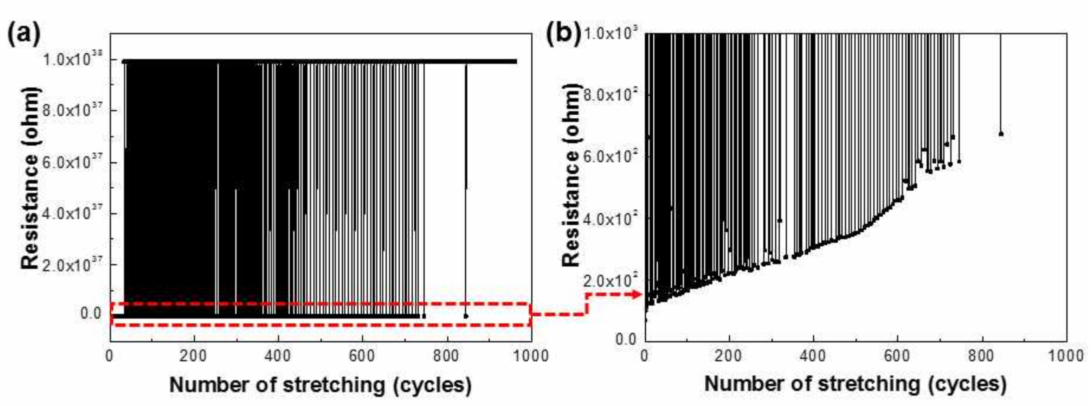

Figure S2. Resistance of the AgNW electrode deposited on a hydroxylated PDMS: (b) shows the resistance values within red squared range in (a) (sample width: 5 mm, length: 10 mm, induced strain: 100%).

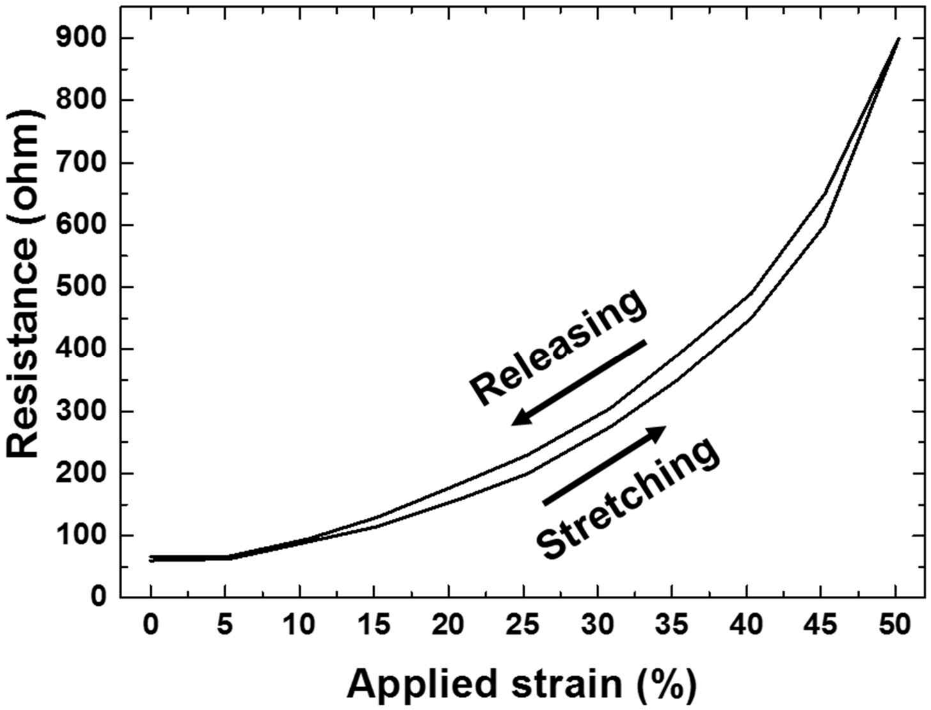

Figure S3. Resistance of the PUU/AgNWs/PDMS electrode measured with stretching up to 50% strain and releasing.
